# Potent inhibitors of malarial *P. Falciparum* protein kinase G: Improving the cell activity of a series of imidazopyridines

**DOI:** 10.1016/j.bmcl.2018.11.039

**Published:** 2019-02-01

**Authors:** Jonathan M. Large, Kristian Birchall, Nathalie S. Bouloc, Andy T. Merritt, Ela Smiljanic-Hurley, Denise J. Tsagris, Mary C. Wheldon, Keith H. Ansell, Peter J. Coombs, Catherine A. Kettleborough, David Whalley, Lindsay B. Stewart, Paul W. Bowyer, David A. Baker, Simon A. Osborne

**Affiliations:** a Centre for Therapeutics Discovery, LifeArc, Accelerator Building, Open Innovation Campus, Stevenage SG1 2FX, UK; b Faculty of Infectious and Tropical Diseases, London School of Hygiene & Tropical Medicine, Keppel Street, London WC1E 7HT, UK

**Keywords:** Malaria, *Plasmodium falciparum*, Protein kinase G, Imidazopyridine, SAR

## Abstract

Development of a class of bicyclic inhibitors of the *Plasmodium falciparum* cyclic GMP-dependent protein kinase (*Pf*PKG), starting from known compounds with activity against a related parasite PKG orthologue, is reported. Examination of key sub-structural elements led to new compounds with good levels of inhibitory activity against the recombinant kinase and *in vitro* activity against the parasite. Key examples were shown to possess encouraging *in vitro* ADME properties, and computational analysis provided valuable insight into the origins of the observed activity profiles.

## Graphical abstract

**Figure:**
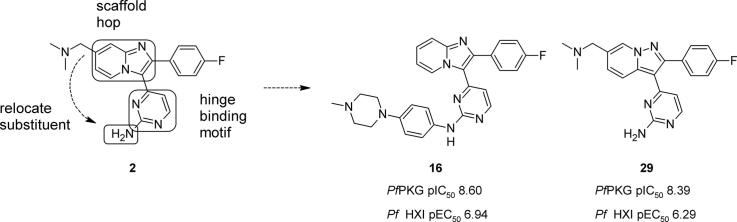

Malaria is one of the most prevalent infectious diseases of the developing world whose causative agent in humans is the protozoan parasite *Plasmodium*, with most deaths caused by the *P. falciparum* species. Despite being largely preventable and treatable, it is currently responsible for almost 0.5 million deaths per year, with young children and pregnant women in sub-Saharan Africa particularly at risk.^1^ The disease also continues to present significant public health policy, social and economic challenges in developing world countries,^2^ with mounting concern about the rapid spread of resistance to current standard antimalarial drugs. The development of structurally and mechanistically novel malaria treatments is urgently required to add to the control tools and advance eradication of the disease.^3^

There is a growing body of evidence to suggest that members of the *P. falciparum* kinome play important roles in multiple stages of the parasite lifecycle.^4^, ^5^ Among these, *P. falciparum* cGMP-dependent protein kinase (*Pf*PKG) is attracting considerable interest as a promising antimalarial drug target. Chemical inhibitors of this enzyme have demonstrated prevention of merozoite egress^6^ and invasion,^7^ gametogenesis,^8^ ookinete motility,^9^ liver cell invasion^10^ and sporozoite motility.^11^ These data continue to suggest that *Pf*PKG remains an appealing drug target for developing new anti-malarial small molecules.

We have recently reported studies on thiazoles^12^ (derived from compound **1**^13^) and on imidazo[4,5-*b*]pyridines as potent and selective inhibitors of *Pf*PKG.^14^ Using the known compound **2**^15^ as a starting point, we prepared new bicyclic analogues which displayed potent inhibitory activity against the enzyme and *in vitro* blood stage anti-parasite activity, good selectivity against human kinases and significant *in vivo* target-driven efficacy.^14^ However, important ADME parameters were thought to remain outside desirable ranges in some cases. Our aim was to investigate key structural motifs in **2** in ways that would address these important physicochemical property considerations, whilst maintaining cell potency and lipophilic ligand efficiency.^16^

We considered that three important initial areas of focus on the structure of **2** would aid the achievement of these objectives. The pyrimidine and its 2-substituent offered opportunities to influence potency and to probe the hinge binding motif against recent crystallographic data^14^ (Figure 1 – A), drawing on our recent studies of a closely related sub-series based on a thiazole core.^12^ A second aspect involved relocating the tertiary amine substituent from its position on the bicyclic scaffold to within an extended aminopyrimidine (Figure 1 – B). Finally, alternative bicyclic cores (Figure 1 – C), some containing additional nitrogen atoms, could contribute to lowering lipophilicity. Our previous experiences with one such related scaffold^17^, ^18^, ^19^, ^20^ suggested that more straightforward synthetic access might also be realised. Herein we report our initial efforts in these areas of work and show how each guided the development of new SAR understanding towards improved *in vitro* profiles as described above.

**Figure 1:**
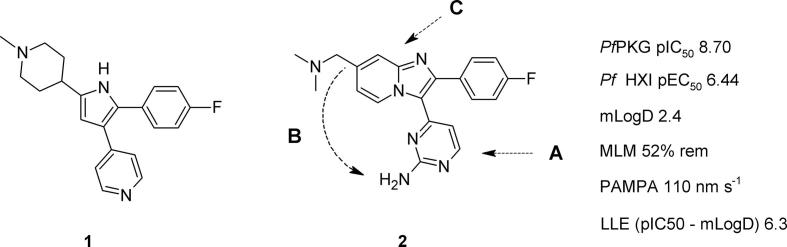
Structures of compounds **1** and **2**, with planned structural changes to imidazopyridines: **A** – probe the hinge binding motif; **B** – re-position the basic centre; **C** – vary the bicyclic scaffold. ADME properties for **2**: mLogD = measured logD; MLM = % remaining after 30 min incubation with mouse liver microsomes; PAMPA = passive permeability.

We first prepared compounds with which to probe the proposed bidentate 2-aminopyrimidine hinge binding motif. The 4-aminopyrimidine isomer **5** was obtained from 4-thiomethyl-6-methylpyrimidine **3**^21^ by means of a three-step conversion to intermediate **4**, transformation of the thiomethyl motif and introduction of the diaminomethyl side chain (Scheme 1). Regioselective iodination of intermediate **6**^22^, followed by mesylation of the alcohol, displacement with dimethylamine and coupling with the appropriate boronic acid gave target compounds **7**–**10** in good yields. A larger aryl piperazine substituent^12^ could be appended to aminopyridine **10** by means of palladium-catalysed arylation, followed by *N*-deprotection to give **11**.

**Scheme 1:**
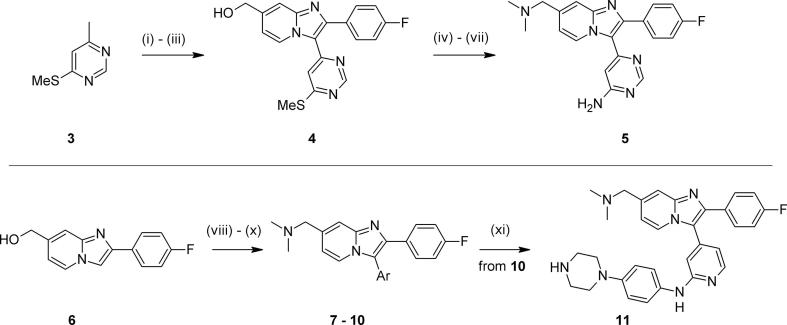
*Reagents and conditions*: (i) LiHMDS, 4-F-C_6_H_4_-CO_2_Me, THF, -10 °C – rt; (ii) Br_2_, AcOH, 0 °C – rt; (iii) 2-aminopyridine-4-methanol, EtOH, 4 Å sieves, 80 °C; (iv) mCPBA, CH_2_Cl_2_, rt; (v) aq NH_3_, THF, microwave, 100 °C; (vi) SOCl_2_, CH_2_Cl_2_, 50 °C; (vii) Me_2_NH, THF, 0 °C – rt; (viii) NIS, CH_2_Cl_2_, MeCN, rt; (ix) MsCl, Et_3_N, CH_2_Cl_2_, 0 °C, then Me_2_NH, THF, rt; (x) PdCl_2_(dppf), ArB(OR)_2_, Cs_2_CO_3_, dioxane, 90 °C; for Ar = 2-aminopyridine only – (xi) Pd(OAc)_2_, Xantphos, 4-bromophenyl-*N*-BOC-piperazine, Cs_2_CO_3_, dioxane, 100 °C, then 4 M HCl in dioxane, rt.

These structural alterations significantly influenced levels of enzyme inhibition (Table 1). The regioisomeric aminopyrimidine **5** was much less active compared to **2** in both biochemical and *in vitro* blood stage anti-parasite activity (data not shown) assays.^23^ The known unsubstituted pyrimidine **7**^15^ showed some recovery against the enzyme. Pyridine **8** was also nearly 40-fold less biochemically active, and other heterocycles such as **9** showed no improvement.^24^ Interestingly, little change was observed on the re-introduction of an amino group in **10**. Introduction of an aryl piperazine motif in **11** resulted in a further drop in potency, in stark contrast to our previous observations on similarly extended aminopyrimidines in the thiazole series.^12^ These data suggested that the 2-aminopyrimidine motif in **2** was likely to provide an optimal interaction with the hinge region of the enzyme.

**Table 1:** Examining the hinge binding motif.

**Figure:**
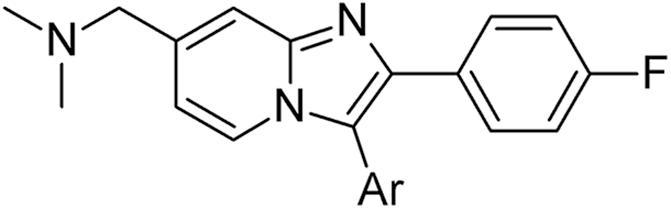

Compound	Ar	*Pf*PKG pIC_50_	LLE^a^
**2**		8.70	6.2

**5**		7.06	4.7

**7**^15^		7.92	5.9

**8**		7.11	4.2

**9**		6.92	5.1

**10**		7.28	5.3

**11**		6.59	*nt*

a *nt* = not tested.

Docking of key examples **2**, **8** and **11** into a recently published *Pf*PKG crystal structure (PDB:5DYK)^25^ provides a possible rationale for understanding these SAR variations. As shown in Figure 2, the bicyclic core of each compound occupies a similar position, directing the 4-fluorophenyl group into a hydrophobic pocket adjacent to the gatekeeper residue (T618), and positioning the dimethylaminomethyl group in **2** and **8** to enable charge interactions with two acidic residues at the solvent interface (E625 and D682). Classical bidentate hydrogen bond formation to hinge residue V621 contributes significantly to the high affinity of **2** and explains the lower affinity of **7** and **8**. However, pyridine **8** requires a larger rotation out of plane from the bicyclic core (32.4° deviation calculated for **8**, 16.5° deviation for **2**), in order to avoid steric clash between hydrogen atoms. Introduction of the larger phenyl piperazine in **11** appeared to allow an edge-face aromatic interaction with Y822 and a relatively long-range charge interaction with E625 at the pocket mouth. Formation of these interactions had been proposed as a route to obtaining a boost in potency in the thiazole series.^12^ In the current case, any benefit is more than offset by realignment to accommodate this bulky group; this results in weakening of other key interactions, among which are hinge binding to V621 and charge interaction between the *N*,*N*-dimethylamine group and E625. It also appears that re-introduction of a bidentate hinge-binding motif in aminopyridine**11** is not sufficient to regain affinity. Though this favours a more coplanar arrangement of the bicyclic core and hinge-binding heterocycle, steric clash between the two rings and the increase in strain energy largely negates any affinity gain. This is reflected in an intermediate calculated dihedral angle (26.2°) for **11**. Hence the current data suggested retaining the amino pyrimidine motif as a more appropriate choice of hinge binding motif, and one which would best allow for effective substituent growth towards the mouth of the binding pocket in future analogues.

**Figure 2:**
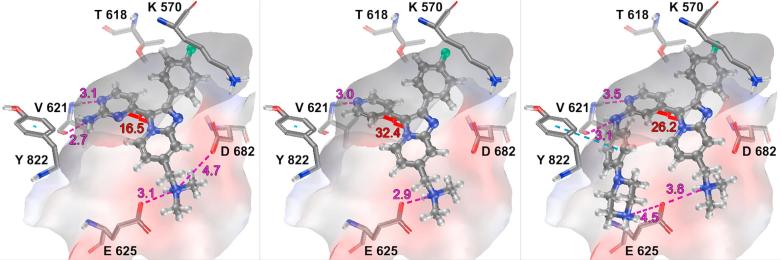
Compounds **2** (left), **8** (centre) and **11** (right) docked into *Pf*PKG, with protein surface coloured by electrostatic potential. Charge-based and H-bonding interactions and distances are shown in purple, the dihedral angles between the bicyclic core and the hinge-binding heterocycle are shown in red, and the edge-face interaction between Y822 and the phenyl ring in compound **11** is shown in light blue.

We turned our attention to the benzylic tertiary amine group attached to the bicyclic core, and hypothesised that re-positioning it to become part of the aminopyrimidine substituent would be beneficial for metabolic stability whilst maintaining potency. In doing so, it was thought that the deletion of both a tertiary amine and a labile benzylic methylene group would remove two likely sites for primary *in vitro* metabolism. In addition, larger aminopyrimidine substituents had previously been shown to provide significant additional *in vitro* potency,^12^ so we wished to explore whether a larger substituent containing a basic centre or other polar group could provide both potency and microsomal stability. The required intermediates **12** or **13** could be assembled using similar chemistry to that described above. The sulfide **12** could be oxidised and easily displaced with more reactive amines, as shown in Scheme 2. For less nucleophilic substrates, alternative acid-catalysed displacement conditions using the chloropyrimidine **13** proved to be more suitable, allowing rapid and efficient preparation of the desired analogues.

**Scheme 2:**
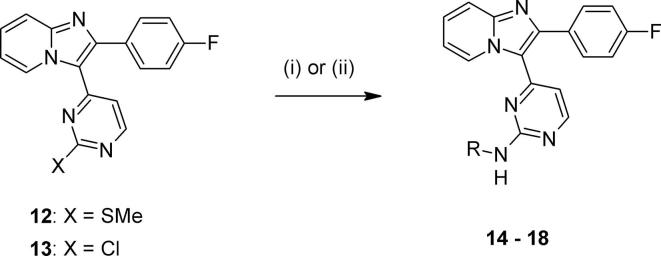
*Reagents and conditions*: for **12** – (i) a) mCPBA, CH_2_Cl_2_, rt; b) RNH_2_, dioxane, 80 °C; for **13** - (ii) RNH_2_, TFA, ^s^BuOH, 110 °C.

We were encouraged to find that early examples such as the simple alkyl amine **14** showed a promising level of *in vitro* potency (Table 2). Conformational constraint in non-basic **15** further supported this approach, improving activity and maintaining a good ADME profile. By extending further *via* a phenyl linker to give aryl piperazine **16**, further significant boosts in both biochemical and anti-malarial activity in a blood stage hypoxanthine incorporation (HXI) cell-based assay were obtained. While metabolic stability for **16** remained reasonably good, other aspects of physicochemistry were likely to be driven by poorer solubility. Adjusting basicity (as in piperidine **17**), or combining with a change in vector (e.g. pyrazole **18**) provided only isolated improvements in ADME properties (eg. solubility for **18**).^26^ The trend of microsomal stability for the aryl aminopyrimidines **16**–**18** was unexpected given their measured logD values, for which there was no convincing explanation. Taken together, these data suggested that this type of aryl linked analogue was in many cases able to provide very good levels of potency, but that a well-balanced overall *in vitro* profile was not easy to locate in compounds lacking the basic substituent on the bicyclic core.

**Table 2:** Relocating the polar bicyclic group.

**Figure:**
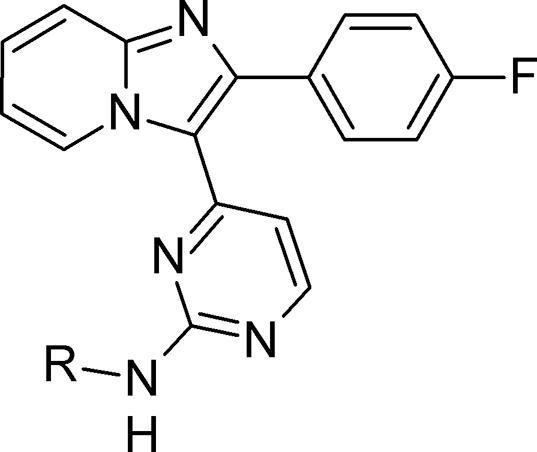

Compound	R	*Pf*PKG pIC_50_	*Pf* HXI pEC_50_^a^	LLE	mLogD	MLM % rem^b^	PAMPA (nm s^−1^)	Kinetic solubility (µM)^a^
**14**		6.07	*nt*	4.3	1.8	62	82	*nt*

**15**		6.88	*nt*	5.1	1.8	84	145	*nt*

**16**		8.60	6.94	4.6	4.0	65	0	9

**17**		8.30	6.80	5.0	3.3	55	0	0

**18**		7.00	6.24	4.3	2.7	37	124	237

a *nt* = not tested.

b % remaining after 30 mins.

To assess the impact of altering the bicyclic core, we selected and prepared several matched-pair analogues in related [6,5-] ring systems. The imidazopyrimidines **19** and **20** could be synthesised from the regioisomer of **3** and an appropriate aminopyrimidine in the same way as described for **5**, with separation of the regioisomeric bicyclic intermediates. Benzimidazole **23** could be prepared by oxidation and displacement of intermediate **21**^23^, followed by reduction of the Weinreb amide in **22** and reductive amination (Scheme 3).

**Scheme 3:**
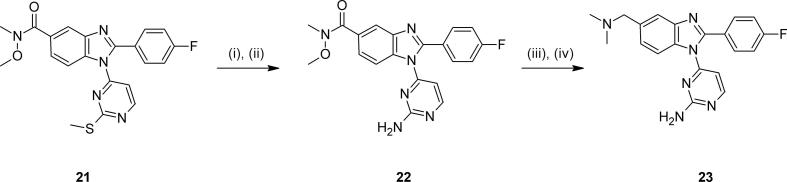
*Reagents and conditions*: (i) H_2_O_2_, Na_2_WO_4_·2H_2_O, AcOH, MeOH, 0 °C – rt; (ii) NH_3_, THF, dioxane, 60 °C; (iii) DIBAL-H, toluene, -78 °C; (iv) Me_2_NH, NaBH(OAc)_3_, CH_2_Cl_2_, THF, rt.

The imidazopyridazine **26** could be accessed by varying a previous chemical approach.^17^ Conversion of bromoketone **24**^15^ to intermediate **25** was followed by oxidation of the thiomethyl group and reaction with ammonia (Scheme 4). Then displacement of the 2-chloro group with the correct amine gave the target **26**. Finally, condensation of the alkynylpyrimidine **27**^27^ with an *N*-aminopyridinium salt gave intermediate **28** and its (undesired) regioisomer. Transformation of the desired regioisomeric product using known chemistry provided the pyrazolopyridine **29**.

**Scheme 4:**
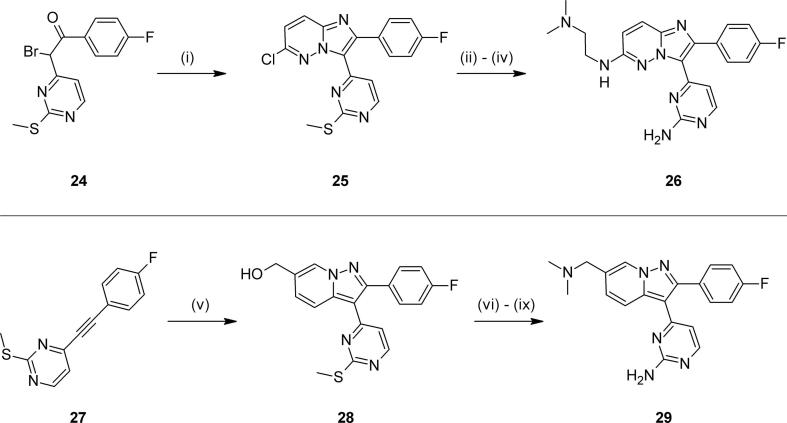
*Reagents and conditions*: (i) 2-amino-6-chloropyridazine, EtOH, 4 Å sieves, 80 °C; (ii) mCPBA, CH_2_Cl_2_, rt; (iii) NH_3_, dioxane, 115 °C; (iv) Me_2_NCH_2_CH_2_NH_2_, DIPEA, NMP, 180 °C; (v) 1-amino-3-(hydroxymethyl)pyridinium iodide, DBU, CH_3_CN, rt; (vi) MsCl, Et_3_N, CH_2_Cl_2_, 0 °C; (vii) Me_2_NH, THF, rt; (viii) H_2_O_2_, Na_2_WO_4_·2H_2_O, AcOH, MeOH, rt; (ix) NH_4_OAc, sealed tube, 120 °C.

These modifications to the bicyclic core once again resulted in considerable variation in *in vitro* activity and ADME profile. The imidazopyrimidine examples **19** and **20** could not in either case match the biochemical activity, anti-parasite activity or lipophilic efficiency of **2**, though both possessed broadly similar ADME profiles (Table 3). Some potency was regained in benzimidazole **23**, as were further improvements in lipophilicity and metabolic stability, though passive permeability was lower. Whilst offering improvements in synthetic tractability and molecular diversity,^17^ imidazopyridazine **26** was significantly less potent and passively permeable. Encouragingly, the pyrazolopyridine **29** regained the best levels of *in vitro* activity and lipophilic efficiency, though further improvements to a promising ADME profile proved difficult to locate in additional examples, particularly with respect to metabolic stability.

**Table 3:** Alternative bicyclic cores.

**Figure:**
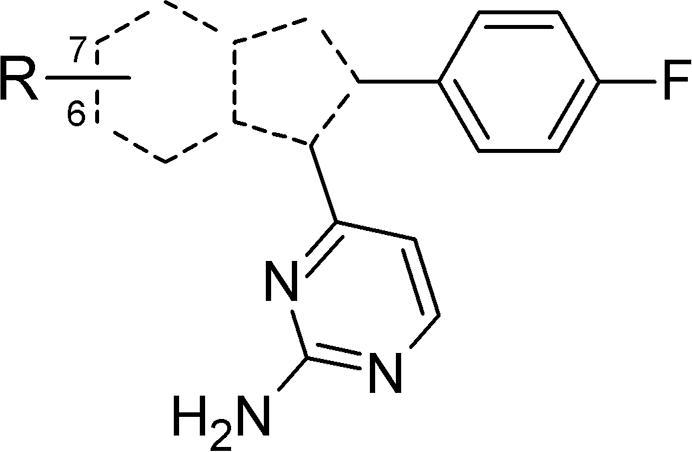

Compound	Core	R	*Pf*PKG pIC_50_	*Pf* HXI pEC_50_^a^	LLE	mLogD	MLM % rem^b^	PAMPA (nm s^−1^)
**2**		7-CH_2_NMe_2_	8.70	6.44	6.3	2.4	52	110

**19**		7-CH_2_NMe_2_	7.26	5.43	5.2	2.1	59	58

**20**		7-CH_2_NMe_2_	7.09	5.39	5.0	2.1	67	118

**23**		7-CH_2_NMe_2_	7.51	*nt*	6.3	1.2	78	32

**26**		6-NH(CH_2_)_2_NMe_2_	6.39	*nt*	4.8	1.6	68	10

**29**		7-CH_2_NMe_2_	8.39	6.29	5.9	2.5	22	185

a *nt* = not tested.

b % remaining after 30 mins.

Computational calculations again suggested a plausible framework by which to understand these data. Each bicyclic system bears a conserved nitrogen atom in the 5-membered ring, but this does not appear to engage the side chain of residue K570 in a bonding interaction in any case. A similar observation was made for a crystal structure of the *P. vivax* PKG enzyme in complex with **2**.^14^ Generation of electronegative field isosurfaces^28^ revealed subtle influences on the hinge binding interaction between the pyrimidine nitrogen atom and the backbone of residue V621. This important interaction was relatively unperturbed for **2** (and also for **29** – data not shown), but appeared to be significantly weakened in the case of benzimidazole **23** (Figure 3). Although a smaller effect seemed to affect the imidazopyrimidines **19** and **20**, higher desolvation penalties due to increased polarity could explain the lowering of affinity for those molecules. The imidazopyridazine **26** showed an electronegative field isosurface very similar to **2** and **29**, but two additional factors may contribute more significantly to its lower affinity. Firstly, the electrostatic repulsion between the nitrogen atoms on the core and pyrimidine ring appears likely to deplanarise that part of the molecule, weakening key interactions in doing so. Secondly, the pendent amine employed may result in less optimal positioning of the charged group, despite increased polarity and flexibility.

**Figure 3:**
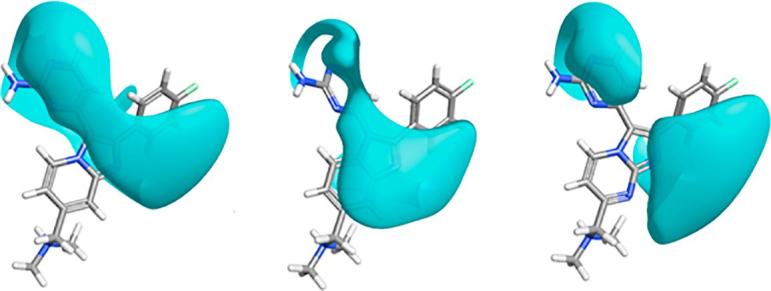
Comparison of ligand electronegative field isosurfaces (calculated using Cresset XED forcefield^28^) for **2** (left), **23** (centre) and **20** (right).

This report has described our initial efforts to develop a series of bicyclic *Pf*PKG inhibitors, based upon a previously described chemical starting point. By focusing on key sub-structural motifs, we have both confirmed and enhanced important SAR and physicochemical trends, resulting in key examples such as **16** and **29**. These two compounds showed good levels of biochemical and *in vitro* anti-malarial activity; **29** in particular possessed equivalent mLogD, improved passive permeability and retained a good level of lipophilic ligand efficiency. Docking and computational analyses provided additional insight into the origins of the observed structure activity relationships. Studies to refine, expand and apply this new knowledge to the preparation of further improved compounds will be reported in due course.
