# Supplementary material for: Potent inhibitors of malarial P. Falciparum protein kinase G: Improving the cell activity of a series of imidazopyridines
Source: Bioorg Med Chem Lett. 2019 Feb 1;29(3):509–14. doi: 10.1016/j.bmcl.2018.11.039 (PMC6355318; doi:10.1016/j.bmcl.2018.11.039)
Supplement: Supplementary data 1 [file mmc1.docx]

**Potent inhibitors of malarial *P. falciparum* protein kinase G: improving the cell activity of a series of imidazopyridines**

Jonathan M. Large,^a,*^ Kristian Birchall,^a^ Nathalie S. Bouloc,^a^ Andy T. Merritt,^a^ Ela Smiljanic-Hurley,^a^ Denise J. Tsagris,^a^ Mary C. Wheldon,^a^ Keith H. Ansell,^a^ Peter J. Coombs,^a^ Catherine A. Kettleborough,^a^ David Whalley,^a^ Lindsay B. Stewart,^b^ Paul W. Bowyer,^b^ David A. Baker,^b^ Simon A. Osborne.^a^

^a^ Centre for Therapeutics Discovery, LifeArc, Accelerator Building, Open Innovation Campus, Stevenage, SG1 2FX, U.K.

^b^ Faculty of Infectious and Tropical Diseases, London School of Hygiene & Tropical Medicine, Keppel Street, London, WC1E 7HT, U.K.

**Supplementary information**

1. **General synthetic chemistry and analysis remarks**
2. **Synthesis and characterisation of key compounds:**
   1. Compound **16**
   2. Compound **29**
3. **ADME assay procedures:**
   1. Measured LogD
   2. Mouse liver microsomal stability
   3. PAMPA permeability
   4. Kinetic solubility
4. **Docking procedure for compounds 2, 8, 11**
5. **General synthetic chemistry and analysis remarks**

All commercial starting materials, reagents and solvents were used without further purification. Flash column chromatography was carried out using either an SP4 or Isolera-4 MPLC system (manufactured by Biotage) with pre-packed KP-silica gel cartridges (supplied by Biotage). ^1^H NMR spectra were obtained using a JEOL ECX400 spectrometer at room temperature; in all cases, NMR data were consistent with the proposed structures. Chemical shifts are given in parts-per-million referenced to residual undeuterated solvent peak, and conventional abbreviations used for multiplicity: e.g. s, singlet; d, doublet; t, triplet; q, quartet; dd, doublet of doublets; br, broad. Analytical mass spectra were recorded using a multi-mode ES+APCI mass spectrometer (6120 quadrupole LCMS manufactured by Agilent). Preparative high pressure liquid chromatography of final compounds was carried out using apparatus made by Agilent. The apparatus is constructed such that the chromatography (column: 19 x 100 mm (5 µm) Prep C-18 XBridge column at a flow rate of 40 ml/min) is monitored by a multi-wavelength UV detector (G1365B manufactured by Agilent) and a multi-mode ES+APCI mass spectrometer (G-1956A, manufactured by Agilent) connected in series, and if the appropriate criteria are met the sample is collected by an automated fraction collector (G1364B manufactured by Agilent). Collection can be triggered by any combination of UV or mass spectrometry, or can be based on time. Typical conditions for the separation process are as follows: the gradient is run over a 10-minute period (gradient at start: 10% methanol and 90% water, gradient at finish: 100% methanol and 0% water). For buffering, either 0.1% trifluoroacetic acid is added to the water (low pH buffer), or 0.1% ammonium hydroxide is added to the water (high pH buffer). It may be necessary or desirable to modify the conditions for a specific compound, for example by changing the solvent composition at the start or at the end, modifying the solvents or buffers, changing the run time, changing the flow rate and/or the chromatography column. For each compound purified, the collected fraction is analysed using four methods: either methanol or acetonitrile as the organic solvent, at either low pH (0.1% TFA) or high pH (0.1% ammonium hydroxide), and if necessary re-purified until ≥ 95% purity at 254 nm was achieved. This criterion was met for all compounds submitted for biological testing. Those obtained as TFA salts were desalted by passing through an Isolute “aminopropyl” NH_2_ cartridge (500 mg or 1 g size, eluting with three column volumes of a 2:1 CH_2_Cl_2_-MeOH solvent mixture), to give the free base before submitting for biological testing or ADME assays.

1. **Synthesis and characterisation of key compounds**
2. Synthesis of compound **16**





(i) NBS, CH_2_Cl_2_, rt; (ii) 2-aminopyridine, dioxane, 60 ^o^C; (iii) ArNH_2_, TFA, ^s^BuOH, 110 ^o^C.

Preparation of intermediate **13**

The ketone starting material was prepared according to *PCT Int. Appl.* WO 2011042389, 2011.

To a solution of the ketone starting material (500 mg, 1.99 mmol) in CH_2_Cl_2_ (12 mL) at room temperature was added N-bromosuccinimide (1.0 eq, 1.99 mmol, 355 mg) and the reaction stirred for 0.5 h. The reaction was concentrated *in vacuo* and the residue dissolved in dioxane (10 mL), treated with 2-aminopyridine (3 eq, 5.98 mmol, 563 mg) and stirred at 60 ^o^C for 18 h. After cooling, the mixture was partitioned between saturated aqueous NaHCO_3_ and EtOAc. The aqueous layer was re-extracted with EtOAc and the combine organic extracts washed with brine, dried (MgSO_4_) and concentrated *in vacuo*. Column chromatography (12 – 100% gradient, EtOAc in petrol) gave the product **13** (395 mg, 61% yield) as a pale yellow solid.

^1^H NMR (400 MHz, DMSO-*d_6_*) δ 9.43 (td, J=1.1, 6.9 Hz, 1H), 8.58 (d, J=5.5 Hz, 1H), 7.79-7.85 (m, 1H), 7.67-7.74 (m, 2H), 7.59 (ddd, J=1.4, 7.0, 8.6 Hz, 1H), 7.29-7.37 (m, 2H), 7.24 (dt, J=1.4, 6.9 Hz, 1H), 7.19 (d, J=5.0 Hz, 1H).

LC-MS (MeOH, pH 1): [M+H]^+^ = 325, 327, rt = 1.84 mins, hplc purity = > 90%.

Preparation of compound **16**

A solution of compound **13** (100 mg, 0.30 mmol) in sec-butanol (1.5 mL) was treated with the aromatic amine (1.1 eq, 0.34 mmol, 65 mg) and trifluoroacetic acid (1.1 eq, 0.34 mmol, 0.03 mL) and the reaction stirred at 110 ^o^C for 18 h. Concentration *in vacuo* and purification by preparative hplc then gave the product **16** (109 mg, 74%) as an off-white solid.

^1^H NMR (400 MHz, DMSO-*d_6_*) δ 9.52 (s, 2H), 8.29 (d, J=5.0 Hz, 1H), 7.72-7.77 (m, 1H), 7.64-7.71 (m, 2H), 7.54 (d, J=8.7 Hz, 2H), 7.43-7.52 (m, 1H), 7.26-7.35 (m, 2H), 7.06 (t, J=6.6 Hz, 1H), 6.90 (d, J=9.2 Hz, 2H), 6.50 (d, J=5.5 Hz, 1H), 3.00-3.14 (m, 4H), 2.42-2.48 (m, 4H), 2.22 (s, 3H).

LC-MS (MeCN, pH 10): [M+H]^+^ = 480, rt = 1.68 mins, hplc purity = > 95%.

1. Synthesis of compound **29**





(i) 1-amino-3-(hydroxymethyl)pyridinium iodide, DBU, CH_3_CN, rt; (ii) MsCl, Et_3_N, CH_2_Cl_2_, 0 ^o^C; (iii) Me_2_NH, THF, rt; (iv) H_2_O_2_, Na_2_WO_4_.2H_2_O, AcOH, MeOH, rt; (v) NH_4_OAc, sealed tube, 120 ^o^C.

Preparation of intermediate **28**

To a cold stirred solution of compound **27**^27^ (6.0 g, 24.6 mmol) and 1-amino-3-(hydroxymethyl)pyridinium iodide (1.3 eq, 31.9 mmol, 8.0 g) in acetonitrile (90 mL) was added DBU (1.3 eq, 31.9 mmol, 4.9 g) and the reaction mixture stirred at room temperature for 3 days. The reaction mixture was diluted with cold water (200 mL) and extracted with EtOAc (2 x 150 mL). The combined organic layers were washed with brine (100 mL), dried (Na_2_SO_4_) and concentrated *in vacuo*. Purification by preparative hplc gave the product **28** (1.6 g, 18%) as off-white solid, together with the undesired regioisomeric by-product (2.7g, 30%) as off-white solid.

^1^H NMR (400 MHz, CDCl_3_) δ ppm 8.52 (s, 1H), 8.45 (d, J=9.2 Hz, 1H), 8.25 (d, J=5.2 Hz, 1H), 7.60-7.57 (m, 2H), 7.38 (d, J=8.8 Hz, 1H), 7.19-7.14 (m, 2H), 6.70 (d, J=5.2 Hz, 1H), 4.78 (d, J=5.6 Hz, 2H), 2.61 (s, 3H), 1.98-1.96 (m, 1H).

LC-MS (MeCN, pH 1): [M+H]^+^ = 367, rt = 2.11 mins, hplc purity = > 95%.

Preparation of intermediate **28A**

To a suspension of compound **28** (250 mg, 0.68 mmol) in CH_2_Cl_2_ (5 mL) was added triethylamine (1.3 eq, 0.89 mmol, 0.13 mL) and the reaction cooled to 0 ^o^C. Then methanesulfonyl chloride (1.5 eq, 1.02 mmol, 0.08 mL) was added and the reaction stirred under nitrogen at 0 ^o^C for 4 h. Further triethylamine (0.65 eq, 0.06 mmol, 0.06 mL) and methanesulfonyl chloride (0.08 eq, 0.51 mmol, 0.04 mL) were added and stirring continued for a further 4 h. Then excess dimethylamine (2M in THF, 10 eq, 6.82 mmol, 3.4 mL) was added, the reaction warmed to room temperature and stirred for 18 h. It was then diluted with water and extracted with CH_2_Cl_2_, the combined organic extracts dried (MgSO_4_) concentrated *in vacuo* and chromatographed (0–10% gradient, 2M NH_3_ / MeOH in EtOAc) to give the product **28A** (216 mg, 80%) as a yellow solid.

^1^H NMR (400 MHz, DMSO-*d_6_*) δ 8.74 (s, 1H), 8.38 (d, J=5.04 Hz, 1H), 8.28-8.34 (m, 1H), 7.58-7.66 (m, 2H), 7.54 (dd, J=1.37, 9.16 Hz, 1H), 7.28-7.38 (m, 2H), 6.78 (d, J=5.50 Hz, 1H), 3.48 (s, 2H), 2.49 (s, 3H), 2.20 (s, 6H).

LC-MS (MeCN, pH 10): [M+H]^+^ = 393, rt = 1.86 mins, hplc purity = > 95%.

Preparation of compound **29**

To a suspension of compound **28A** (216 mg, 0.55 mmol) in methanol (6 mL), were added hydrogen peroxide (4.0 eq, 2.2 mmol, 0.067 mL), acetic acid (5.0 eq, 2.74 mmol, 0.16 mL) and sodium tungstate dihydrate (0.3 eq, 0.16 mmol, 54 mg) and the reaction stirred under nitrogen at room temperature for 4 h. Saturated NaHCO_3_ (aq) as added and the mixture extracted with CH_2_Cl_2_. The combined organic extracts were dried (MgSO_4_) and concentrated *in vacuo*. The product was then dissolved in CH_2_Cl_2_ (1 mL) and excess CS_2_ (0.25 mL) and sodium sulfate added. After stirring overnight under nitrogen, LC-MS analysis indicated removal of the unwanted amine N-oxide by-product. Saturated NaHCO_3_ (aq) was carefully added and the mixture extracted with CH_2_Cl_2_. The combined organic extracts were dried (MgSO_4_) and concentrated *in vacuo* to give a mixture of the sulfoxide and sulfone (163 mg).

LC-MS (MeCN, pH 10): [M+H]^+^ = 410 (sulfoxide: rt = 1.40 minutes), 426 (sulfone: rt = 1.63 minutes), hplc purity of mixture = > 90%.

A portion of this material (108 mg) was treated with excess NH_4_OAc (1 g) and heated in a sealed vessel at 120 ^o^C for 4 h. After cooling, DMSO (1 mL) and water (0.5 mL) were added, and the mixture filtered through cotton wool and purified by preparative hplc to give the product **29** (7 mg) as a white solid.

^1^H NMR (400 MHz, DMSO-*d_6_*) δ 8.68 (s, 1H), 8.46 (d, J=9.16 Hz, 1H), 8.01 (d, J=5.50 Hz, 1H), 7.57-7.71 (m, 2H), 7.41 (dd, J=1.37, 9.16 Hz, 1H), 7.28-7.38 (m, 2H), 6.62 (s, 2H), 6.18 (d, J=5.04 Hz, 1H), 3.49 (br. s., 2H), 2.21 (s, 6H).

LC-MS (MeCN, pH 10): [M+H]^+^ = 363, rt = 1.30 minutes, hplc purity = > 95%

1. **ADME assay procedures**
   1. Measured logD

LogD measurements were carried out using the shake flask method. Compound was diluted from 10 mM DMSO stock solution into an eppendorf containing equal amounts of octanol and phosphate buffered saline (PBS) to give a final concentration of 100 μM. The tubes were shaken for 12 hours, centrifuged at 10000 rpm for 10 minutes and samples taken from the octanol and PBS layers. The samples from both layers were analysed in triplicate by LC-MS/MS (Agilent Technologies G6410 series, triple quadrupole with MM-ESI ion source) using optimised multiple reaction monitoring (MRM) scans and a standard column gradient on an Acquity UPLC BEH C8 1.7 μm column, running acetonitrile and water with 0.05% Acetic Acid as the mobile phase. The ratios of areas of the peaks were used to calculate the LogD in accordance with the equation:

LogD = Log_10_(Area-TL/Area BL)

- 1. mouse liver microsomal turnover

Microsomal turnover data was obtained using mouse liver microsomes (MLM) obtained from BD Biosciences. The compounds are pre-incubated at 37 °C for 5 minutes with the microsomes and the reaction was initiated by adding an equal volume of NADPH generating solution, also obtained from BD Biosciences. The final compound concentration in the incubation is 1 μM, and the microsomal protein concentration is 0.2 mg/mL. A sample is taken at t=0 and quenched with 2x volume of ice-cold methanol containing an internal standard reference compound (carbamazepine). The reaction is agitated at 37 °C for 30 minutes, when a further sample was taken and quenched in an identical fashion. The samples were centrifuged at 10000 rpm for 10 minutes and the supernatant taken for analysis in triplicate by LC-MS/MS (Agilent Technologies G6410 series, triple quadrupole with MM-ESI ion source) using optimised multiple reaction monitoring (MRM) scans and a standard column gradient on an Acquity UPLC BEH C8 1.7μm column, running acetonitrile and water with 0.05% acetic acid as the mobile phase. The % turnover is obtained by calculating the percentage difference of the peak areas, normalised to the internal standard, at t=0 and t=30. Verapamil is used as the standard compound for this assay.

- 1. PAMPA permeability

PAMPA assays were carried out using a 96 well BD Biosciences pre-coated PAMPA plate with 0.4 μm polyvinylidene fluoride filter plate, precoated with structured layers of phospholipids. The compounds were run in triplicate on the same plate with at a specified concentration between 200 μM and 50 μM solution which is known to be below the previously determined kinetic solubility, the final solution having a PBS:DMSO ratio of 98:2. The compound solution was placed in the lower ‘Donor’ well of the plate and the upper ‘Acceptor’ well of the plate was filled with PBS:DMSO 98:2 buffer containing no compound following which the plates was assembled then incubated for 5 hours at room temperature. After incubation the Donor and Acceptor wells were sampled into flat UV bottomed plates and a six-fold 1:2 dilution series created for each compound starting at the appropriate top concentration for each compound. The UV absorbance for these solutions was read across 230-400 nm at 1 nm intervals using a TECAN Safire II plate reader and a suitable UV wavelength chosen around the UV maximum of each compound. This was used to calculate the concentration in the Donor and Acceptor wells for each compound. The compound permeability P_app_ (nm s^-1^) and % mass retention was then calculated for each compound in accordance with the following equations:

P_app_ (nm s^-1^) =10000000 {-ln[-C_A_(t)/C_eq_]}/[A*(1/V_D_+1/V_A_)*t]

C_eq_ = [C_D_(t)* V_D_+ C_A_(t)*V_A_]/(V_D_+V_A_)

% mass retention = 1 – [C_D_(t)*V_D_ + C_A_(t)*V_A_]/(C_0_*V_D_)]

A = Filter Area, V_D_ = Donor Well Volume, V_A_ = Acceptor Well Volume, t = incubation time (secs), C_A_(t) = Acceptor concentration at time t, C_D_(t) = Donor concentration at time t, C_0_ = Initial Donor concentration

- 1. Kinetic solubility

The kinetic solubility was measured by diluting a small amount of 10 mM DMSO stock into PBS pH7.4 in a filtration plate at a target concentration of 200 μM giving a final solution composition of 98:2 PBS:DMSO. Each compound was run in triplicate on the same plate with two standard compounds, verapamil and ketoconazole, included per plate. The filtration plate was shaken at 500 rpm for 90 minutes and then filtered under vacuum. The filtrate was sampled and diluted with a DMSO:PBS mixture in a flat bottomed UV plate to give a solution with a composition of PBS:DMSO 80:20. A dilution series for each compound was then created in flat bottomed UV plates in PBS:DMSO 80:20 with concentrations 200 μM, 100 μM, 50 μM, 25 μM, 12.5 μM & 6.75 μM. The UV absorbance for these solutions was read across 230-400 nm at 1 nm intervals using a TECAN Safire II plate reader and a suitable UV wavelength chosen around the UV maximum of each compound. This was used to calculate the concentration in the filtrate for each compound, and hence amount remaining in solution after 90 minutes which is reported as the kinetic solubility.

1. **Docking procedure for compounds 2, 8, 11**

Docking studies were carried out using the Schrodinger suite. Compounds were prepared using the LigPrep utility to generate a low energy conformation, with ionisation state at pH 7.4 assigned using Epik. The protein (PDB:5DYK) was prepared using the Protein Preparation Wizard with default parameters, including assignment of bond orders, tautomer and ionisation states, addition of hydrogen atoms, removal of water molecules, optimisation of H-bonding networks and restrained minimisation using the OPLS2005 forcefield. Residues A823 and E824 immediately preceding a stretch of amino acids not visible in the model were removed because of their poor fit to the electron density, to avoid their unreliable positioning confounding the docking results. The grid for docking was generated using a 12-angstrom inner box and 32-angstrom outer box centred on the ATP binding site, with H-bond constraints to the peptide backbone of hinge residue V621. Docking was carried out using GlideSP (rigid receptor, fully flexible ligand) with default parameters, including strain correction terms and outputting up to 5 poses. The lowest energy poses were consistent between ligands, building confidence in the binding mode hypothesis.

For further details see Cheeseright, T.; Mackey, M.; Rose, S.; Vinter, A. *J. Chem. Inf. Model.* **2006**, *46*, 665.
